# Redox-responsive targeted gelatin nanoparticles for delivery of combination wt-p53 expressing plasmid DNA and gemcitabine in the treatment of pancreatic cancer

**DOI:** 10.1186/1471-2407-14-75

**Published:** 2014-02-08

**Authors:** Jing Xu, Amit Singh, Mansoor M Amiji

**Affiliations:** 1 Department of Pharmaceutical Sciences, School of Pharmacy, Northeastern University, 360 Huntington Avenue, Boston, MA 02115, USA

**Keywords:** Thiolated gelatin, Wt-p53 expressing plasmid DNA, Gemcitabine, Pancreatic Adenocarcinoma, Apoptosis

## Abstract

**Background:**

Pancreatic adenocarcinoma is one of the most dreaded cancers with very low survival rate and poor prognosis to the existing frontline chemotherapeutic drugs. Gene therapy in combination with a cytotoxic agent could be a promising approach to circumvent the limitations of previously attempted therapeutic interventions.

**Method:**

We have developed a redox-responsive thiolated gelatin based nanoparticle system that efficiently delivers its payload in the presence of glutathione-mediated reducing intra-cellular environment and could be successfully used for site-specific wt-p53 expressing plasmid DNA as well as gemcitabine delivery by targeting epidermal growth factor receptor (EGFR). Efficacy studies were performed in subcutaneous human adenocarcinoma bearing SCID beige mice along with molecular level p53 plasmid and apoptotic marker expression by PCR and western blot for all study groups.

**Results:**

Efficacy studies demonstrate an improved *in vivo* targeting efficiency resulting in increased transfection efficiency and tumor growth suppression. In all the treatment groups, the targeted nanoparticles showed better anti-tumor activity than their non-targeted as well as non-encapsulated, naked therapeutic agent counterparts (50.1, 61.7 and 77.3% tumor regression by p53 plasmid alone, gemcitabine alone and in combination respectively). Molecular analysis revealed a higher mRNA expression of transfected p53 gene, its corresponding protein and that the tumor cell death in all treatment groups was due to the induction of apoptotic pathways.

**Conclusions:**

Gene/drug combination treatment significantly improves the therapeutic performance of the delivery system compared to the gene or drug alone treated groups. Anti-tumor activity of the thiolated gelatin loaded wt-p53 plasmid or gemcitabine-based therapy was attributed to their ability to induce cell apoptosis, which was confirmed by a marked increase in mRNA level of proapoptotic transcription factors, as well as, protein apoptotic biomarker expression and significant decrease in the anti-apoptotic transcription factors.

## Background

Pancreatic cancer is the fourth leading cause of cancer-related deaths with an estimated 45,220 newly diagnosed cases and an expected death of 38,460 patients in 2013 in United States alone [1]. Only 10% of the diagnosed patients have a resectable stage of tumor that could be potentially cured by surgical procedures [2]. Even in the case of surgically resectable tumor, incidence of aggressive metastasis resurgence often leads to development of resistance to conventional chemo and radiation therapy. Despite several advancements in diagnosis, surgical methods, chemo and radiation therapy, the prognosis of the disease remains poor with less than 5% five-year survival rate. Poor prognosis can be largely attributed to late diagnosis of the disease where most of the patients present with advanced stages localized or metastatic cancer growth. Patients with advanced stage localized pancreatic tumor show a 6–10 months median survival while those suffering from a metastatic form of the disease only have a 3–6 months median survival [3].

Chemotherapy still remains the most popular approach for treatment of advanced stage localized or metastatic pancreatic adenocarcinoma and gemcitabine (2′-2′-difluorodeoxycytidine) has been used as the front-line therapeutic drug for this purpose. However, gemcitabine in combination with other therapeutic agents such as platinum analogues [4,5], anti-metabolites [6-8] or topoisomerase inhibitors [9,10], has failed to elucidate any improvement in the therapeutic outcome or survival rate. FLOFIRINOX recently has been proposed as an alternative to gemcitabine-based therapy due to an improved median overall survival of 11.1 months compared to 6.8 months [11]. However, there still lacks an overall consensus on the optimal therapeutic regimen for pancreatic cancer where the majority of the chemotherapeutic clinical trials have been terminated in their phase II or III stage due to unfavorable or insignificant outcomes. A recent review on analysis of clinical trials on second-line treatment in locally advanced or metastatic pancreatic cancer also summarizes that neither FLOFIRINOX nor gemcitabine-based regimen have been able to provide a standard of care against the disease [12]. There is, therefore, an urgent need to revisit the therapeutic approach and design novel strategies to overcome this disease.

Every case of pancreatic cancer can be characterized by nearly 63 genetic mutations that could be classified into 12 core signaling pathways [13], providing diversity in physico-chemical signature of a tumor. Mutation in p53 tumor suppressor gene is a well-established fact associated with pancreatic cancer [14], which not only results in promotion of tumorigenesis but also affects the apoptotic mechanism of tumor cell death and is further associated with induction of chemo-resistance [15,16]. Gemcitabine or 5-fluorouracil resistance in pancreatic cancer has been attributed to an altered expression of apoptosis-regulating genes of the Bcl-2 family [17], which are regulated by p53 level in the cell. *In vitro* evidence clearly suggests that introduction of wild-type p53 gene into the pancreatic cancer cells increases their sensitivity to gemcitabine therapy [18]. Gene delivery, however, is extremely challenging due to instability of genetic material and lack of targeting and is often achieved with the aid of a suitable delivery system. Also, another mechanism for gemcitabine resistance in pancreatic cancer cells is due to lower intracellular drug uptake attributed to lack of nucleoside transporter [19], which warrants for alternative delivery methods. Combination therapy of p53 gene along with gemcitabine encapsulated in a delivery vehicle therefore would be a promising approach to augment therapeutic benefits and overcome challenges associated with pancreatic cancer treatment.

We have previously reported long-circulating redox-responsive thiolated type B gelatin (SH-Gel-PEG) that shows tremendous potential as stimuli-responsive gene delivery vehicle [20-26], such that the thiol crosslinks of the nanoparticles could be disrupted in the glutathione-mediated reducing intracellular environment of the cell resulting in payload release and transgene expression. We recently developed an EGFR-targeted thiolated gelatin-based delivery system that could deliver wild-type p53 (wt-p53) gene efficiently in Panc-1 human adenocarcinoma cells. EGFR-targeted thiolated gelatin nanoparticles loaded with wt-p53 plasmid showed rapid uptake and plasmid release, enhanced gene expression and subsequent higher protein levels causing apoptosis induction and cell death [26]. Qualitative and quantitative biodistribution studies in Panc-1 tumor bearing mice showed a significantly higher tumor accumulation of the targeted long circulating thiolated gelatin nanoparticles (SH-Gel-PEG-peptide) compared to SH-Gel-PEG and SH-Gel nanoparticles [27].

In the present work, we have not only evaluated the *in vivo* transfection efficiency and therapeutic efficacy of different gelatin nanoparticles, but also show that these nanoparticles could be used for delivery of gemcitabine *in vitro* and *in vivo*, thus allowing the possible combination of gene and drug therapy against pancreatic cancer. Subcutaneous animal models are most commonly used tumor models in preclinical research since they are fast to develop, easy to characterize and present reasonable heterogeneity and complexity of the actual human tumors [28]. Subcutaneous pancreatic adenocarcinoma model was therefore developed in severe combined immunodeficient (SCID) beige mice using Panc-1 cells for all the *in vivo* studies.

## Methods

### Preparation of wt-p53 plasmid loaded gelatin nanoparticles

Thiolated gelatin was synthesized and purified using an established method that conjugates 2-iminothiolane to primary amine groups on type B gelatin [23,25]. Lyophilized purified thiolated gelatin was used for nanoparticle preparation and encapsulation of plasmid by desolvation method developed and optimized in our lab [23,25,29]. Typically, 1% (w/v) thiolated gelatin solution was prepared in deionized distilled water at 37°C and pH was adjusted to 7 using 0.2 M NaOH. 1 mg plasmid DNA was gently mixed in the gelatin solution followed by slow addition of chilled ethanol with continuous stirring at 600 rpm. Gelatin nanoparticles are formed when the solvent composition changes to 75% hydro-alcoholic solution following which 0.5 mL 8% (v/v) glyoxal solution was added drop-wise to crosslink the thiol group. The particles were purified and concentrated by tangential flow filtration, freeze-dried and stored at 4°C until used.

SH-Gel-PEG and SH-Gel-PEG-peptide nanoparticles were prepared by a method described before [23,29]. Briefly, freeze-dried nanoparticles (10 mg/mL) were suspended in 0.1 M phosphate buffer (pH 7.4) and incubated with methoxy-PEG-succinimidylcarbosyl methyl ester (mPEG-PEG-SCM, MW 2000 Da) or maleimide-PEG-SCM (MAL-PEG-SCM, MW 2000 Da) for 2 h at room temperature with slow stirring to form SH-Gel-PEG and SH-Gel-PEG-MAL particles respectively. The particles were purified by ultra-centrifugation at 18,800 g for 30 min (Beckman Coulter Optima™ LE-80 k; rotor 70Ti; Brea, CA), washed twice in deionized water and freeze-dried. SH-Gel-PEG-MAL particles (10 mg/mL) were suspended in 0.1 M phosphate buffer (pH 6.5) with 10% weight of 12 amino acid EGFR binding peptide flanked with four glycine spacer and a terminal cysteine (i.e., ***Y-H-W-Y-G-Y-T-P-Q-N-V-I***-G-G-G-G-C) for 6 h at room temperature to facilitate binding of the sulfhydryl group of cysteine to maleimide group on PEG. The peptide modified nanoparticles were purified by ultra-centrifugation at 16,000 rpm for 30 minutes, washed twice in deionized water, freeze-dried and stored at 4°C until used. The physico-chemical properties, plasmid loading efficiency and stability of the particles were characterized, details of which have been published elsewhere [26]. The typical average size of gelatin nanoparticles was found to be between 130-230 nm with SH-Gel being the smallest in size (~ 130 nm). PEG modification of the SH-Gel particles increased the size to nearly 180 nm and subsequent peptide functionalization further increased the size to nearly 230 nm. The average surface charge of the nanoparticles was found to be around -20 mV and the p53 gene loading efficiency was found to be around 95%.

### Preparation of gemcitabine loaded gelatin nanoparticles

10 mg base form of gemcitabine was dissolved in 5 mL methanol with 100 mg succinimidyl 3-[2-pyridyldithio]-propionate) (SPDP) at 80°C under reflux for 48 hours. The reaction was monitored by thin-layer chromatography (TLC) (*R*_*f*_ 0.67 (CHCl_3_/MeOH, 8:2)). Solvent was removed *in vacuo* with rotary evaporator IKA RV10 at 60°C, and the residue was purified by silica gel chromatography (200 mL, CHCl_3_ and 200 mL CHCl_3_/MeOH, 9:1) to give gemcitabine-SPDP. UV spectrometer was used to monitor elute at λ = 268 nm.

Purified gemcitabine-SPDP was dried *in vacuo* and then dissolved in 1 mL dimethyl sulfoxide (DMSO). For conjugation with gemcitabine-SPDP, thiolated gelatin (10 mg/mL) was dissolved in 0.1 M PBS/EDTA (100 mM sodium phosphate, 150 mM NaCl, 1 mM EDTA, 0.02% sodium azide, pH 7.5). Gemcitabine-SPDP was added to thiolated gelatin solution and stirred overnight at room temperature. Formed gemcitabine-gelatin disulfide conjugates were dialyzed against DI water overnight and then purified polymers were used for nanoparticle synthesis. The conjugation of gemcitabine-SPDP and gemcitabine-gelatin were confirmed by reverse phase HPLC, using a C_18_ column (Thermo-Fisher Scientific, MA), with the UV detector set at 268 nm. The mobile phase was composed of 20% of MeOH/H_2_O (5:5) and 80% 0.5 M ammonium acetate solution. The elution was performed by isocratic flow and flow rate was 1 mL/min. Standard curve was established with pure gemcitabine and release of drug was determined based on standard curve. Gemcitabine loaded gelatin nanoparticles were synthesized following the protocol similar to one used for p53 gene loaded nanoparticle synthesis (described above). The size and charge measurement of gemcitabine loaded SH-Gel, SH-Gel-PEG and SH-Gel-PEG-peptide nanoparticles were found to be consistent with that observed for p53 gene loaded nanoparticles. The average particle size was found to be between 130-230 nm and the average surface charge for all the different nanoparticle systems was found to be around -20 mV.

*In vitro* release of gemcitabine from the nanoparticles was performed in the presence of proteolytic enzyme (0.2 mg/mL) and glutathione to mimic the intracellular (5 mM) and extracellular (0.1 mM) environment in the tumor [30]. The drug release studies were carried out at 37°C with PBS solution as control. 20 mg of gemcitabine-loaded nanoparticles were weighed into microcentrifuge tubes and dissolved in 1.5 ml of buffer containing glutathione and/or protease. Samples were incubated in temperature controlled shaker and 0.5 ml of supernatant was drained at specified intervals (15, 30, 45, 60, 120, 240 and 360 minutes). Sink conditions were maintained by replacing an equal volume of release medium each time. Collected samples were centrifuged at 13,000 rpm for 15 min, filtered through 0.2 μm filters and analyzed by reverse phase HPLC, using a C_18_ column using assay condition described above. Additional file 1: Figure S1 shows the drug release profile of SH-Gel, SH-Gel-PEG and SH-Gel-PEG-peptide nanoparticles.

### Subcutaneous pancreatic tumor model development

Panc-1 human pancreatic adenocarcinoma cells were obtained from American Type Culture Collection ATCC, Manassas, VA) (Manassas, VA) and were grown in DMEM media supplemented with 10% FBS and 1% Pen-Strep. Animal handling and procedures were performed according to an approved protocol by Northeastern University, Institutional Animal Care and Use Committee (NU-IACUC) and the Radiation Safety Committee within the office of Environmental Health and Safety. Six weeks old female SCID Beige mice, weighing approximately 20 g, were purchased from Charles River Laboratories (Wilmington, MA) and were used for efficacy studies.

To inoculate subcutaneous tumors, animals were mildly anesthetized by inhalation of 2% Isoflurane (St. Joseph, MO) in 100% oxygen and approximately 3 million Panc-1 cells in 100 μl of PBS and Matrigel mixture (1:1) was injected subcutaneously into the left flanks of female SCID Beige mice. Tumors were allowed to grow and reach a palpable volume and during this period, the animals were monitored for food/water intake, body weight and any signs of discomfort. Any animals that seemed lethargic were sacrificed.

### *In vivo* nanoparticle administration and dosing schedule

The animals were randomized into different treatment groups for efficacy studies when the tumor volume reached 200 mm^3^. The dosing schedule and treatment groups for different formulations of wt-p53 alone, gemcitabine alone and wt-p53/gemcitabine in combination have been outlined in Additional file 1: Figure S2. The plasmid treatment group mice were each administered with 20 μg plasmid at day 0, 2 and 4. From the 12 mice per treatment group, 3 mice were euthanized at day 7 and 18 for in vivo transfection analysis while remaining 6 mice were sacrificed after completion of the study (day 33). Similarly, mice receiving gemcitabine treatment were intravenously administered with 4 doses of free or formulated drug at a dose of 5 mg/kg at day 0, 7, 14 and 21.

The wt-p53 plasmid and gemcitabine combination efficacy study was performed where plasmid loaded particles were dosed at day 0, 2 and 4 followed by gemcitabine loaded particle administration at day 5, 12, 19 and 26. *In vitro* studies of gelatin nanoparticle loaded p53 gene transfection followed by assessment of its expression and effect on downstream apoptosis markers revealed that apoptotic activity is maximum 96 hour post-transfection [26]. The first dose of gemcitabine was therefore administered at day 5 when the apoptotic effect of p53 gene would have taken effect. All doses for efficacy studies were administered to the animals intravenously via tail vein injection. Tumor volumes were measured and recorded every 3 days for all treatment groups and all the mice were sacrificed at the completion of the study (day 33). The mice were sacrificed by isoflurane inhalation followed by cervical dislocation, tumor mass was weighed and flash frozen in liquid nitrogen for analysis of protein, mRNA and apoptotic markers.

### Quantitative transfection efficiency and downstream apoptosis marker evaluation

*In vivo* gene transfection efficiency was evaluated by quantitative polymerase chain reaction (qPCR). For mRNA extraction, tumors were excised and stored in RNA*later*® (Invitrogen, Carlsbad, CA) at 4°C overnight and then -20°C for long-term storage. mRNA of tumor samples was extracted using Powergen 125 tissue homogenizer (Fisher Scientific, Waltham, MA) and TRIzol® Reagents, PureLink® RNA Mini Kit (Invitrogen, Carlsbad, CA). Extracted RNA was measured with Nano-Drop® 2000 (Thermo-Scientific, Wilmington, DE). cDNA was synthesized from 2 μg of extracted mRNA with SuperScript® III First-Strand Synthesis SuperMix Kit (Invitrogen, Carlsbad, CA). 2 μL of synthesized cDNA and LightCycler® 480 SYBR Green kit (Roche, Indianapolis, IN) were used for qPCR in LightCycler® 480 and analyzing mRNA levels of Flag-p53 and corresponding downstream transcription factors. Primer sequences (Additional file 1: Table S1) for p53, Bax, Bcl-2, β-actin, DR5, Apaf-1, PUMA, caspase 3 and caspase 9 were synthesized in Eurofins MWG Operon (Huntsville, AL). All the results for gene expression have been calculated and reported relative to the control group.

### Qualitative transfection efficiency and downstream apoptosis marker evaluation

Proteins were extracted from tumors using Total Protein Extraction Kit (Millipore, Billerica, MA) and Powergen 125 tissue homogenizer (Fisher Scientific, Waltham, MA). Tissue lysate samples were analyzed for total protein concentration using BCA assay (Pierce, Rockford, IL, USA). 50 μg of total protein extract was run on pre-cast 4-20% sodium dodecyl sulfate-polyacrylamide gel electrophoresis (SDS-PAGE) system at 200 V for 30 minutes. Subsequently, protein bands on gel were transferred onto PVDF membrane by iBlot® Dry Blotting System (Invitrogen, Carlsbad, CA). Membrane was blocked with 5% milk in Tween®-containing Tris buffer saline (TBS-t) for 1 hour at room temperature. Membrane was cut and incubated with 1:1,000 dilution of primary rabbit β-actin antibody, 1:500 primary rabbit cleaved PARP antibody, 1:500 primary rabbit cleaved caspase 3 (Cell Signaling Technology Inc., Danvers, MA) or 1:1000 dilution of primary mouse monoclonal anti-FLAG®M2 antibody (Sigma-Aldrich, St. Louis, MO) separately overnight at 4°C. Membranes were then washed three times with TBS-t and incubated with 1:2,000 dilutions of secondary anti-rabbit or anti mouse horse-radish peroxidase-conjugated IgG(Cell Signaling Technology Inc., Danvers, MA) in TBS-t for 1 hour at room temperature. After rinsing excess antibody with TBS-t and water, 4 ml ECL substrate (Pierce, Rockford, IL, USA) was added and mixed with membranes for 5 minutes, which is cleaved by peroxidase to give a chemiluminescent product. The membranes were visualized using Kodak Digital X-ray Specimen (DXS) System. β-actin was used as protein loading control.

### Terminal deoxynucelotidyl transferase dUTP nick end labeling (TUNEL) analysis

TUNEL analysis was performed on the tumor sections to confirm the DNA fragmentation as a result of activation of apoptotic signaling cascade. Excised tumors were embedded in frozen section medium (Richard-Allan Neg 50, Thermo Scientific, Waltham, MA), flash frozen in liquid nitrogen, and stored at -80°C until use. Embedded tumors were thawed to -20°C, cryo-sectioned into 10 μm thick sections using the Microm® HM550 cryostat (MICROM International GmbH, Germany), and mounted onto glass slides (SuperFrost Plus®, Thermo Scientific, Waltham, MA). Sections were air dried at room temperature and then stored at -20°C. DeadEnd™ Fluorometric TUNEL System (Promega, Madison, WI) was used for tissue staining. After staining, tissues were mounted with Fluoromount-G (Southern Biotech, AL), covered with a coverslip, sealed with nail polish and imaged by Olympus BX61 microscope.

### Statistical analysis

All the statistical analysis was performed using Prism 5.0 software (Graph Pad Software Inc., San Diego, CA). Results were expressed as mean ± SD of the at least three independent experiments. Data was analyzed by Student’s t-test or one way ANOVA followed by Bonferroni’s post hoc analysis for multiple comparisons. Differences were considered statistically significant at p < 0.05.

## Results

### *In vitro* studies show enhanced activity and targeting efficiency

The wt-p53 loaded thiolated gelatin nanoparticles were in the size range of 150-250 nm as confirmed by light scattering and scanning electron microscopy analyses. *In vitro* studies show high transfection efficiency and subsequent increased production of p53 protein in Panc-1 cells that results in triggering of downstream apoptotic pathways, inducing cell death [26]. We simultaneously synthesized gemcitabine conjugated to gelatin according to the scheme shown in Additional file 1: Figure S3, which resulted in particles in the size range of 150-250 nm with a negative surface charge similar to the values obtained for wt-p53 plasmid-loaded nanoparticles. HPLC analysis reveals 24.9% gemcitabine loading efficiency in the particles, which could be released by treatment with 0.2 mg/mL protease and 5 mM DTT treatment, emulating the cancer intracellular environment [31]. *In vitro* cytotoxicity assessment in Panc-1 cells show IC50 values for free drug, Gem-SPDP, Gem-Gel, Gem-Gel-PEG and Gem-Gel-PEG-peptide nanoparticles to be 129.9 ± 23.87, 8.39 ± 1.79, 24.76 ±7.99, 20.08 ± 6.97 and 17.08 ± 2.32 μM respectively, confirming that the drug-loaded gelatin nanoparticles shows improved cytotoxicity compared to the free drug.

### Gelatin particles loaded with wt-p53 gene show efficient transfection ability and anti-tumor activity *in vivo*

**Figure 1:**
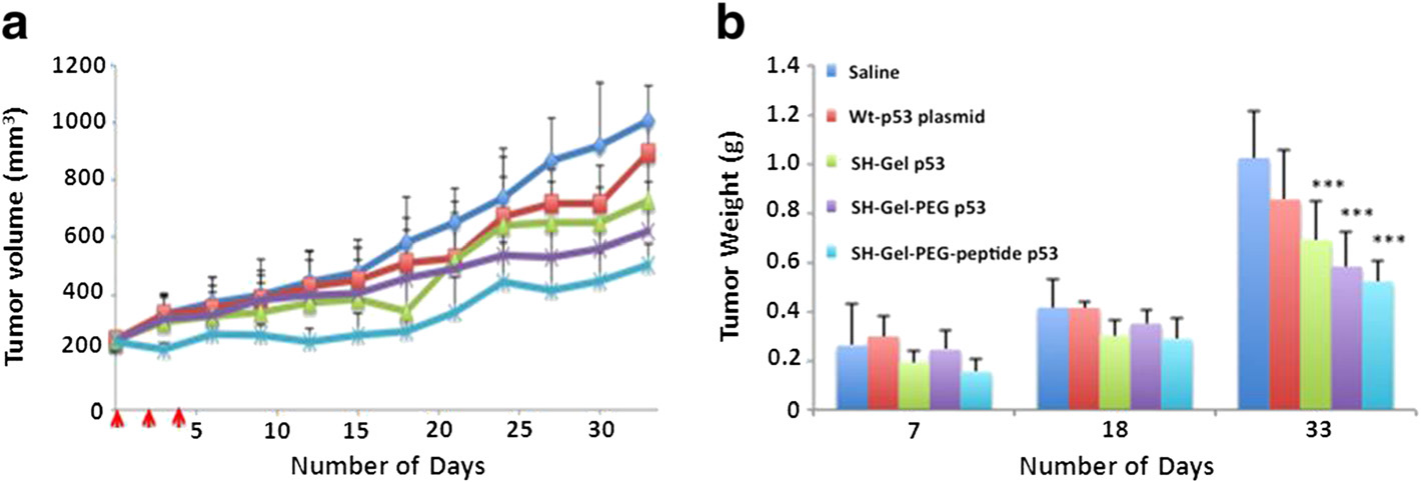
**In vivo anti-tumor activity of wt-p53 loaded gelatin nanoparticles. (a)** Volume change as a function of time showing 27.9, 38.3* and 50.1%** reduction in tumor growth on day 33 for wt-p53 loaded SH-Gel, SH-Gel-PEG and SH-Gel-PEG-peptide nanoparticle treated tumor respectively. **(b)** Tumor weights on day 7, 18 and 33. Results are presented as mean ± SD (n = 3 for day 7 and 18; n = 6 for day 33) (* p < 0.05; ** p < 0.01; *** p < 0.001).

Subcutaneous human pancreatic adenocarcinoma (Panc-1) bearing female SCID beige mice (n = 12) were intravenously dosed with wt-p53 naked and gelatin encapsulated plasmid (20 μg/dose) at day 0, 2 and 4 and tumor volume was monitored as a function of time till the end of the study (day 33). Also, 3 mice from each treatment group were sacrificed at day 7 and 18 to measure tumor weight, gene expression and analysis of downstream apoptotic markers. Plasmid loaded SH-Gel-PEG-peptide and SH-Gel-PEG nanoparticles produced tumor growth inhibition of 50.1% (p < 0.01) and 38.3% (p < 0.05) respectively compared to control group, confirming that wt-p53 gene administration show tumor growth suppression (Figure 1a). On the contrary, naked as well as SH-Gel loaded plasmid shows little anti-tumor activity suggesting that targeting and long-circulating characteristics are essential for enhanced intra-tumor localization of the nanoparticles. Weight of the tumors obtained from mice of different treated groups after day 7, 18 and 33 (Figure 1b) do not show remarkable difference at day 7 and 18 but were found to be significantly decreased compared to control at day 33.

**Figure 2:**
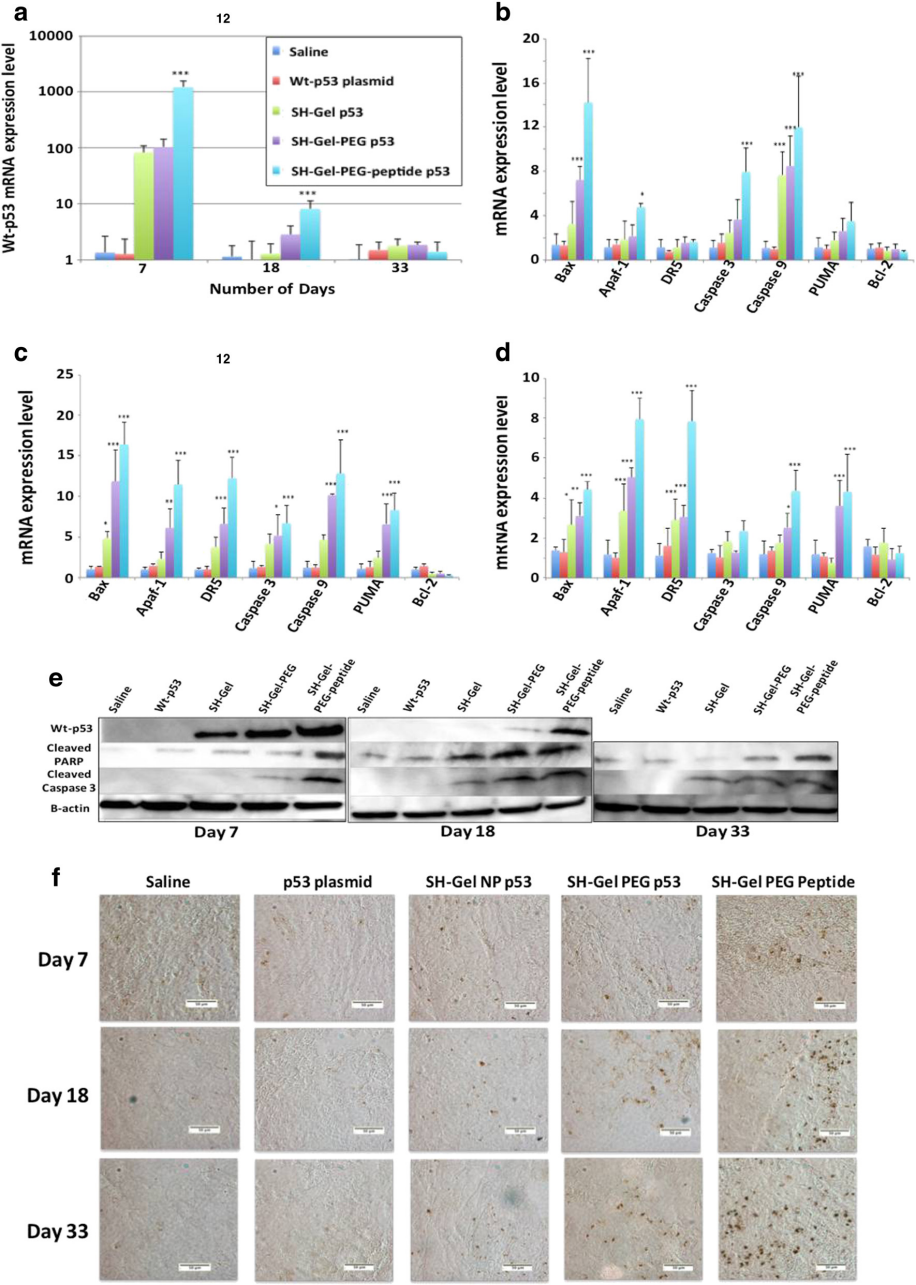
**Transfection efficiency and apoptotic activity of wt-p53 loaded gelatin nanoparticles. (a)** p53 mRNA expression in tumors. Results are presented as mean ± SD (n = 3 for day 7 and 18; n = 6 for day 33). mRNA expression level for downstream apoptotic markers after **(b)** day 7, **(c)** day 18 and **(d)** day 33. (n = 3 for day 7 and 18; n = 6 for day 33) (*p < 0.05; **p < 0.01; ***p < 0.001). **(e)** Western blot analysis for p53, cleaved PARP, cleaved caspase 3 and β-actin protein expression in treated tumors. **(f)** TUNEL analysis of apoptotic activity in the wt-p53 treated tumors. Sections of tumor tissues imaged after treatment for 7, 18 and 33 days for TUNEL positive (brown) cells. All images were acquired at 20× with scale bar of 50 μm.

The expression of wt-p53 and downstream apoptotic markers in the tumors after day 7, 18 and 33 were confirmed quantitatively by qPCR (Figure 2a-d) and qualitatively by western blot analysis (Figure 2e). SH-Gel-PEG-peptide treated tumors show remarkably higher levels of p53 mRNA (p < 0.001) as well as protein after 7 and 18 days of treatment while SH-Gel-PEG treated tumors also show higher p53 mRNA expression compared to control. Naked plasmid on the other hand did not show any change in mRNA level, confirming that a delivery system is essential for successful transfection and gene activity *in vivo*. Further, no significant p53 mRNA or protein expression was observed in any of the treatment groups (Figure 2a,e).

Expression of p53 gene has been long known to induce stable growth arrest and apoptosis in cancer cells [15,16] and it is therefore pertinent to assess the mRNA and protein levels of downstream apoptotic markers to validate the activity of p53 protein. To assess the effect of exogenous wt-p53 on apoptotic pathway, downstream pro-apoptotic transcription factors Bax, DR5, Apaf-1, PUMA, caspase3 and caspase9 and anti-apoptotic Bcl-2 were also analyzed with qPCR (Figure 2B-D). The results clearly indicate a marked increase in all the pro-apoptotic transcription factors upon treatment with SH-Gel-PEG-peptide while SH-Gel-PEG and SH-Gel treated tumors show a moderate increase in expression. These results were consistent with western blot analysis for cleaved caspase 3 and cleaved PARP in tumors, signature protein indicators confirming induction of apoptosis (Figure 2e). Both proteins levels were increased with transfection of wt-p53, where highest protein levels were observed with SH-Gel-PEG-peptide p53 treatment on day 18. These studies demonstrated that expression of wild-type p53 triggered apoptotic pathway in tumors through up-regulation of pro-apoptotic transfection factors and down-regulation of anti-apoptotic transfection factors. TUNEL stained imaging of tumors xenografts was also performed for visual analysis of distribution of apoptotic cells. Images show that SH-Gel-PEG-peptide treated tumors have the highest number of TUNEL positive cells (Figure 2f). SH-Gel PEG p53 and SH-Gel NP p53 were also able to increase the apoptotic cell population compared to saline and naked plasmid control.

### Gemcitabine conjugated gelatin nanoparticles show increased cytotoxicity and tumor growth inhibition

**Figure 3:**
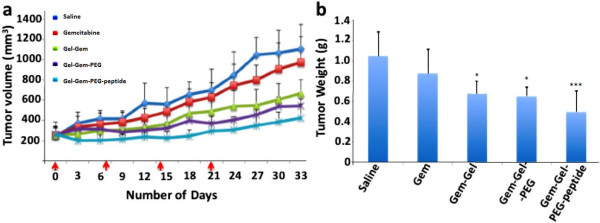
**In vivo anti-tumor activity of gemcitabine conjugated gelatin nanoparticles. (a)** Volume change as a function of time showing 39.4*, 50.7** and 61.7%*** reduction in tumor growth on day 33 for Gem-Gel, Gem-Gel-PEG and Gem-Gel-PEG-peptide nanoparticle treated tumor respectively. **(b)** Tumor weights were measured at day 33. Results are presented as mean ± SD (n = 6; *p < 0.05; **p < 0.01; ***p < 0.001).

Prior studies for pancreatic cancer treatment involving gene therapy in combination with gemcitabine have administered drug dose as high as 100 mg/kg/week [32] but improved efficacy due to intervention of nano-delivery systems has significantly reduced the required dose [33-35]. In this study, we administered 4 weekly doses of 5 mg/kg to the subcutaneous human pancreatic adenocarcinoma (Panc-1) bearing female SCID beige mice followed by tumor volume measurement every 3 days till day 33. Gem-Gel-PEG-peptide treatment group inflicted maximum tumor growth inhibition (61.7%, p < 0.001) while Gem-Gel-PEG and Gem-Gel treatment groups caused 50.7 (p < 0.01) and 39.4% (p < 0.05) growth inhibition (Figure 3a). Interestingly, gemcitabine drug in solution did not show any statistically significant tumor inhibition effect, emphasizing the improved efficacy of the drug by virtue of the delivery vehicle. Tumor weight measured at the completion of the efficacy study (day 33) corroborated the trend indicated by tumor volume measurement where EGFR-targeted nanoparticle treated tumor shows minimum mass (p < 0.001) compared to non-targeted and non-PEG gelatin nanoparticle treated tumors (Figure 3b).

**Figure 4:**
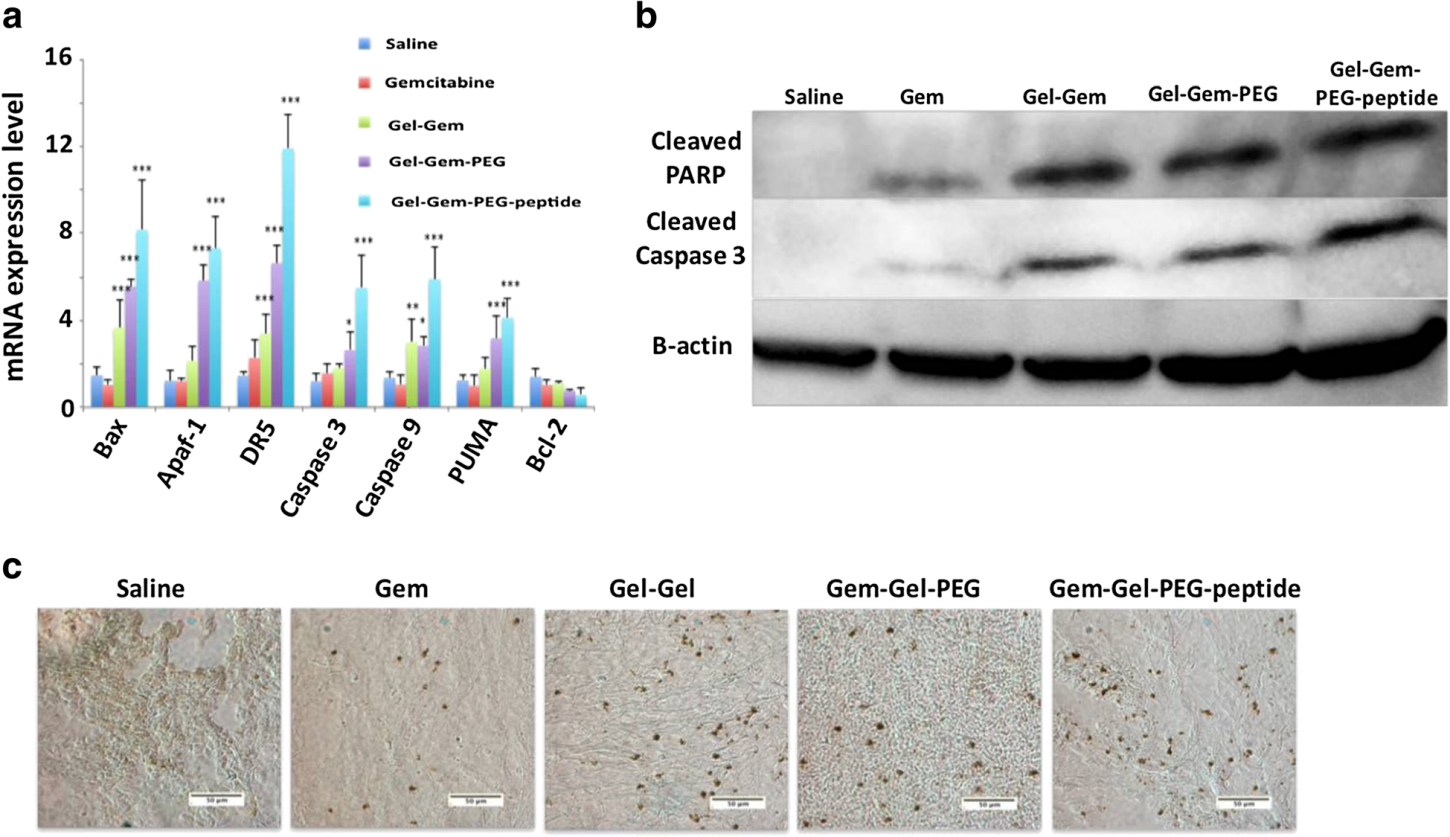
**Assessment of apoptosis mediated cell death induction by gemcitabine conjugated gelatin nanoparticles. (a)** mRNA expression profile for transcription factors in the apoptotic pathway (n = 6, *p < 0.05; **p < 0.01; ***p < 0.001). **(b)** Western blot analysis for cleaved PARP, cleaved caspase 3 and β-actin protein expression in treated tumors. **(c)** TUNEL stained images of treated tumors indicating DNA fragmentation (brown stain). All images were acquired at 20× with scale bar of 50 μm.

The mechanism for gemcitabine action is based on DNA damage that triggers the apoptotic pathway. Effect of gemcitabine treatment on apoptosis was evaluated quantitatively by measurement of mRNA levels of transcription factors (Bax, Bcl-2, DR5, Apaf-1, PUMA, caspase 3 and caspase 9) by qPCR and qualitatively by protein analysis. Targeted nanoparticles significantly increased mRNA expression of all the pro-apoptotic transcription factors (p < 0.001) while Gem-Gel-PEG and Gem-Gel nanoparticles were also able to increase the expression of these transcription factors, but to lesser extent (Figure 4a). Besides, anti-apoptotic transfection factor Bcl-2 mRNA level was slightly decreased with nanoparticle treatments. Protein analysis by western blot validated these results, where both cleaved caspase 3 and cleaved PARP show a moderate increase with nanoparticles treatment (Figure 4b) where the highest protein level was observed in Gem-Gel-PEG-peptide treated tumors. Gemcitabine induced DNA damage was also assessed by TUNEL staining, which showed maximum TUNEL-positive cells in targeted nanoparticle treatment group compared to non-targeted and non-PEG nanoparticle treatment (Figure 4c). These studies demonstrated that active targeted delivery of gemcitabine into tumors showed enhanced anti-tumor activity by successfully triggering the apoptotic pathway.

### Combination wt-p53/gemcitabine treatment shows remarkable inhibition in tumor growth

**Figure 5:**
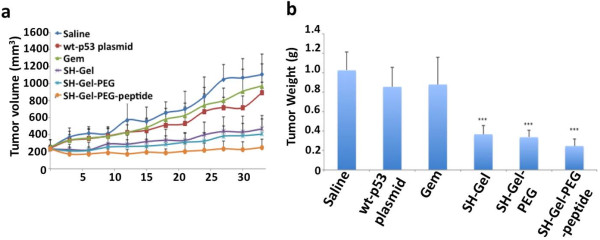
***In vivo *efficacy assessment of wt-p53/gemcitabine combination treatment. (a)** Volume change as a function of time showing 57.6***, 63.3.7*** and 77.3%*** reduction in tumor growth on day 33 for SH-Gel, SH-Gel-PEG and SH-Gel-PEG-peptide nanoparticle treated tumor respectively. **(b)** Tumor weights measured at day 33. Results are presented as mean ± SD (n = 6; ***p < 0.001).

We studied the efficacy of wt-p53 and gemcitabine combination treatment in subcutaneous human pancreatic adenocarcinoma bearing female SCID beige mice. The tumor bearing animals (n = 6) first received 3 doses of wt-p53 plasmid (20 μg plasmid/dose) encapsulated in thiolated, non-targeted and EGFR-targeted thiolated gelatin nanoparticles at day 0, 2 and 4. Gemcitabine conjugated to gelatin was administered in 4 weekly doses (5 mg/kg) at day 5, 12, 19 and 24. Tumor volume measurement during the course of the study showed maximum tumor growth inhibition by targeted nanoparticle based combination treatment (77.3%, p < 0.001) while non-targeted and non-PEG modified systems showed 63.3 and 57.6% (p < 0.001) growth inhibition (Figure 5a) compared to control. Importantly, combination treatment by targeted system proved to be most effective in tumor growth inhibition compared to individual wt-p53 gene (Figure 1a) or gemcitabine (Figure 4a) treatment using same delivery system (50.1 and 61.7% respectively).

The increased tumor growth inhibition suggests a synergistic contribution of the two therapeutic moieties in the combination treatment. A recent report also suggests that p53 gene transfection in pancreatic cancer sensitizes the cells to gemcitabine therapy, which could be an alternative mechanism involved in improving the therapeutic outcome [36]. However, since p53 gene alone shows significant tumor growth suppression in the absence of gemcitabine, the possibility of a synergistic effect seems more likely. Tumor weight measurements performed at the end of the study (day 33) also showed a significant decrease in tumor mass of treated group compared to the control, further confirming that gene-drug combination treatment results in tumor growth inhibition (Figure 5b). The tumors obtained from mice treated with EGFR-targeted system show minimum mass, which was consistent with the trend obtained from tumor volume measurements.

**Figure 6:**
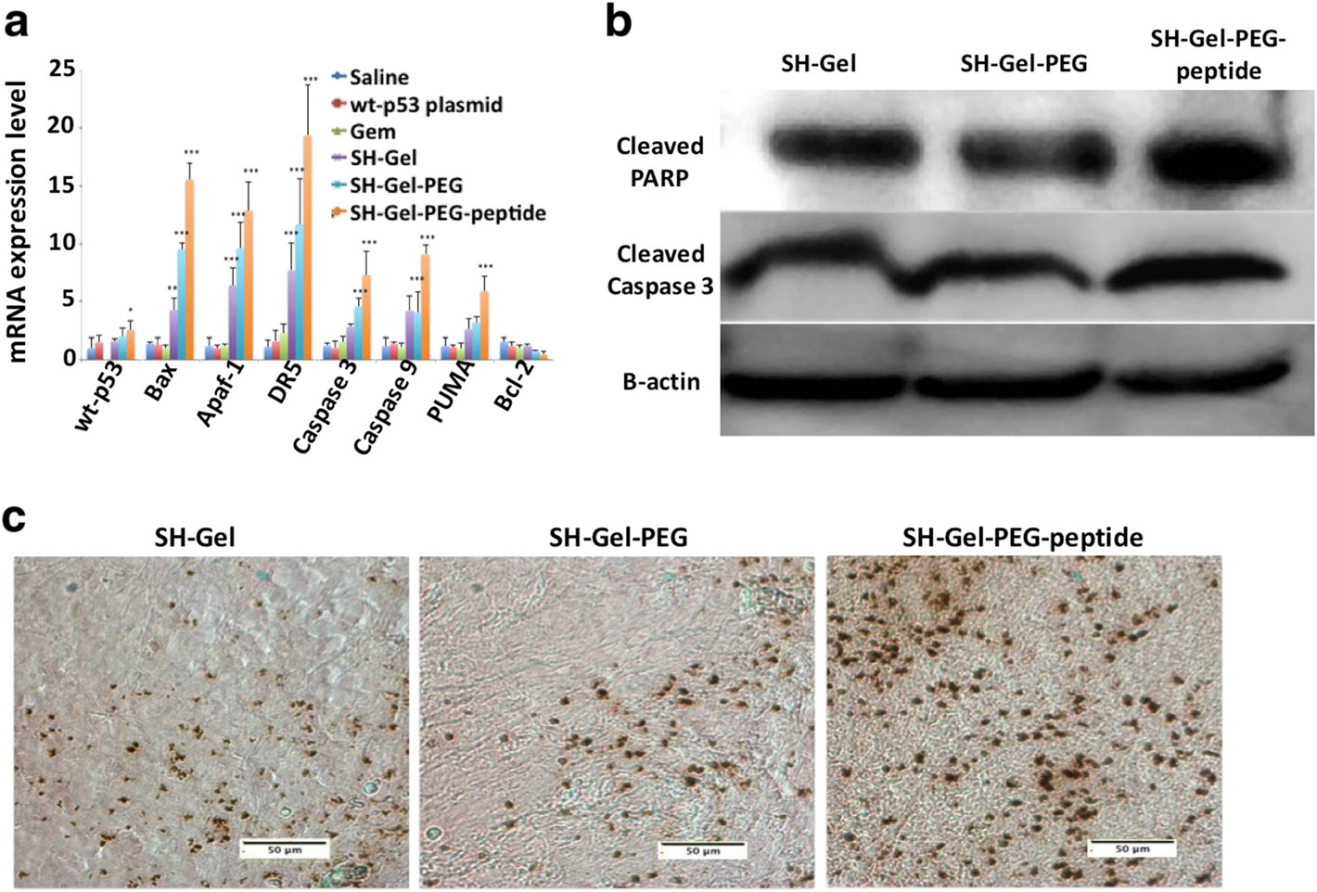
**Transfection and apoptosis induction efficiency of combination treatment. (a)** mRNA expression profile for p53 and transcription factors in the apoptotic pathway. (n = 6, *p < 0.05; **p < 0.01; ***p < 0.001). **(b)** Western blot analysis for cleaved PARP, cleaved caspase 3 and β-actin protein expression in treated tumors. **(c)** TUNEL stained images of treated tumors indicating DNA fragmentation (brown stain). All images were acquired at 20× with scale bar of 50 μm.

Wt-p53 mRNA expression levels were checked for the treatment groups quantitatively by qPCR, which show higher expression in tumors treated with targeted nanoparticles but the expression level in general was low for all treatment groups (Figure 6a). Similar expression profile was also observed in tumors harvested after day 33 from mice treated with wt-p53 encapsulated in gelatin nanoparticles (Figure 2a), suggesting loss of plasmid activity over time. Western blot analysis for p53 protein in tumor treated with combination therapy did not show detectable signal, further implying the low mRNA and subsequent protein level. Analysis of mRNA levels of the transcription factors for downstream apoptosis pathway reveals that all the pro-apoptotic markers are up regulated in tumors treated with combination therapy using targeted gelatin nanoparticles while anti-apoptotic marker Bcl-2 is largely down regulated (55%) (Figure 6a). SH-Gel and SH-Gel-PEG nanoparticle treated tumors, on the other hand, show a moderate increase in the pro-apoptotic markers compared to the control. Western blot analysis of cleaved caspase 3 and cleaved PARP as downstream apoptotic proteins was performed on samples from tumors treated with combination therapy of different nanoparticles (Figure 6b). EGFR targeted gelatin nanoparticles treated tumors show moderately increased caspase 3 and significantly increased PARP levels compared to those treated with SH-Gel and SH-Gel-PEG. TUNEL staining performed on sections of the tumor also confirmed that EGFR-targeted nanoparticle treated tumor section show highest population of cells undergoing apoptosis (brown TUNEL positive cells) compared to those treated with SH-Gel and SH-Gel-PEG (Figure 6c). These observations clearly indicated that treatment with wt-p53, followed by gemcitabine has triggered the apoptotic pathway to a higher extent in tumors through up-regulation of pro-apoptotic transfection factors and down-regulation of anti-apoptotic transfection factors.

## Discussion

Chemotherapy alone does not show significant improvement in prognosis of pancreatic cancer and novel combination therapies are therefore actively pursued to achieve synergistic therapeutic benefits. Gene therapy involving p53 gene replacement in combination with a genotoxic drug such as gemcitabine is a promising direction since the drug induces DNA damage [37] while p53 plays a key role in cellular response to such damage [15]. Delivery of a gene or drug to its site of action *in vivo* however is an extremely challenging task due to presence of several physico-chemical and physiological barriers. Formulation of a therapeutic moiety in a delivery vector is therefore of utmost importance to prevent drug/gene degradation or rapid clearance by the reticulo-endothelial system (RES) and provide a favorable pharmacokinetic profile. The surface of the delivery vehicle could be also leveraged to design a site-specific receptor targeted system to improve tumor accumulation ability thereby enhancing the therapeutic benefit of the payload.

We have developed a long-circulating thiolated gelatin-based redox responsive system that delivers the payload in response to the intracellular glutathione-mediated reducing environment [25]. We have previously demonstrated its successful application in gene delivery *in vitro*[20,26] as well as favorable systemic biodistribution *in vivo*[24,27]. Recently, we have successfully formulated a gelatin nanoparticle-based system for delivery of gemcitabine, the frontline drug used in treatment of pancreatic cancer. In the present work, we study the efficacy effect of gelatin loaded with wt-p53 gene and gemcitabine independently as well as in combination in subcutaneous human pancreatic adenocarcinoma (Panc-1) bearing female SCID beige mice.

One of the main aims of this work was to study the therapeutic benefit from targeting the nano-delivery system *in vivo*. We designed 3 thiolated gelatin nanoparticle systems, SH-Gel, SH-Gel-PEG and EGFR-targeted SH-Gel-PEG-peptide that were either loaded with wt-p53 gene or gemcitabine. Biodistribution studies with these particles have previously revealed that mean residence time (MRT) of these particles is significantly higher and that addition of PEG corona did not necessarily increase it further. Presence of EGFR-targeting peptide on the surface however did result in a higher nanoparticle accumulation in the tumor indicating potential therapeutic advantage [27]. Targeted nanoparticle system was therefore tested against parent therapeutic component as well as non-targeted and non-PEG coated nanoparticles to confirm the indication from biodistribution profile. Another important aim of the study was to investigate the efficacy result of individual gene or drug treatment and compare it with therapeutic outcome of gene/drug combination treatment.

Therapeutic efficacy of targeted nanoparticles were compared to the non-targeted and non-PEG modified gelatin nanoparticles for wt-p53 treatment alone, gemcitabine treatment alone and the combination treatment and the targeted particles outperformed all the other treatment groups in the respective therapeutic regimen. They showed much higher tumor growth inhibition capability resulting in lowest tumor weight for each category of treatment regimen, confirming that active targeting of the tumor *in vivo* has promising therapeutic potential. Both p53 and gemcitabine show tumor growth suppression activity owing to their induction and regulation of apoptosis and thus we checked the mRNA levels of the pro and anti-apoptotic transcription factor as well as downstream apoptotic protein expression in the treated tumors. All the therapeutic procedures adopted in the study consistently showed an increased upregulation of pro-apoptotic transcription factors and apoptotic protein marker in tumors treated with targeted system compared to control and other gelatin nanoparticle-based formulation. TUNEL staining of the tumor sections gave a visual evidence of increased apoptotic cells consistent with the trends observed by mRNA and protein levels assessment. All these results clearly indicate that targeting strategy is important to enhance the drug delivery efficiency of the nano-systems and should be actively pursued to improve the performance of the formulation.

Cytotoxic drug administration in combination with gene therapy is a promising approach for the treatment of pancreatic cancer especially since drugs alone have failed to give a promising outcome in disease prognosis. Our results conclusively demonstrate improved therapeutic efficacy when wt-p53 gene is administered into the tumor bearing mice in combination with gemcitabine. Targeted thiolated gelatin system presents an improved tumor growth suppression activity in combination therapy (77.3%) compared to wt-p53 (50.1%) or gemcitabine (61.7%) treatment alone. Pro-apoptotic transcription factor and apoptotic protein marker expression is also significantly increased in the combination treatment and the TUNEL stained tumor sections show a marked increased in the apoptotic cell population. These results cement the hypothesis that otherwise persistent pancreatic cancer tumors would potentially show an improved response to a gene/drug combination therapy, when administered within a delivery vector to obtain a favorable pharmacokinetic profile.

## Conclusions

Redox-responsive EGFR-targeted thiolated gelatin-based delivery system was designed for wt-p53 expressing plasmid or gemcitabine encapsulation and subsequent treatment of pancreatic cancer in subcutaneous tumor bearing SCID mice. Gene/drug combination treatment groups show significantly greater anti-tumor activity compared to the gene or drug alone treatments groups and among the combination treatment groups, targeted nanoparticles exhibit superior efficacy. Molecular level assessment confirms that tumor growth inhibition for all the treatment groups take effect by induction of apoptosis.

## Abbreviations

ANOVA: Analysis of variance; Apaf1: Apoptosis protease activation factor 1; ATCC: American type culture collection; Bax: Bcl-2 associated X; Bcl-2: B-cell lymphoma 2; DMEM: Dulbecco’s modified eagles medium; DR5: Death receptor 5; EGFR: Epidermal growth factor receptor, IgG, Immunoglobulin G; PARP: Poly ADP ribose polymerase; PEG: Polyethylene glycol; SCID: Severe combined immunodeficiency; PUMA: p53 upregulated modulator of apoptosis; TUNEL: Terminal deoxynuclotidyl transferase dUTP nick end labeling.

## Competing interests

The authors declare no conflict of interests.

## Authors’ contributions

JX, AS and MMA contributed to design of experiments. JX performed the experiments and analyzed data. Manuscript was written by AS and the entire work was supervised by MMA. All authors have read and approved the manuscript prior to submission.

## Pre-publication history

The pre-publication history for this paper can be accessed here:

http://www.biomedcentral.com/1471-2407/14/75/prepub

## Supplementary Material

Additional file 1 **Figure S1.** Gemcitabine drug release profile from SH-Gel, SH-Gel-PEG and SH-Gel-PEG-peptide with protease and glutathione in PBS (n = 3, Mean ± SD). **Figure S2.** Treatment groups and dose schedule used for **(A)** p-53 administration, **(B)** gemcitabine administration and **(C)** p53-gemcitabine combination adminstration in subcutaneous Panc-1 tumor bearing mice. **Figure S3.** Scheme demonstrating the steps involved in synthesis of gemcitabine conjugated thiolated gelatin. **Table S1.** Primer sequences used for qPCR analysis of wt-p53, Bax, Apaf-1, DR5, β-actin, Bcl-2, Caspase 3, Caspase 9 and PUMA. Click here for file
