# Supplementary material for: Redox-responsive targeted gelatin nanoparticles for delivery of combination wt-p53 expressing plasmid DNA and gemcitabine in the treatment of pancreatic cancer
Source: BMC Cancer. 2014 Feb 8;14:75. doi: 10.1186/1471-2407-14-75 (PMC3927583; doi:10.1186/1471-2407-14-75)
Supplement: Additional file 1 — Figure S1. Gemcitabine drug release profile from SH-Gel, SH-Gel-PEG and SH-Gel-PEG-peptide with protease and glutathione in PBS (n = 3, Mean ± SD). Figure S2. Treatment groups and dose schedule used for (A) p-53 administration, (B) gemcitabine administration and (C) p53-gemcitabine combination adminstration in subcutaneous Panc-1 tumor bearing mice. Figure S3. Scheme demonstrating the steps involved in synthesis of gemcitabine conjugated thiolated gelatin. Table S1. Primer sequences used for qPCR analysis of wt-p53, Bax, Apaf-1, DR5, β-actin, Bcl-2, Caspase 3, Caspase 9 and PUMA. [file 1471-2407-14-75-S1.docx]

**ADDITIONAL FILE 1**


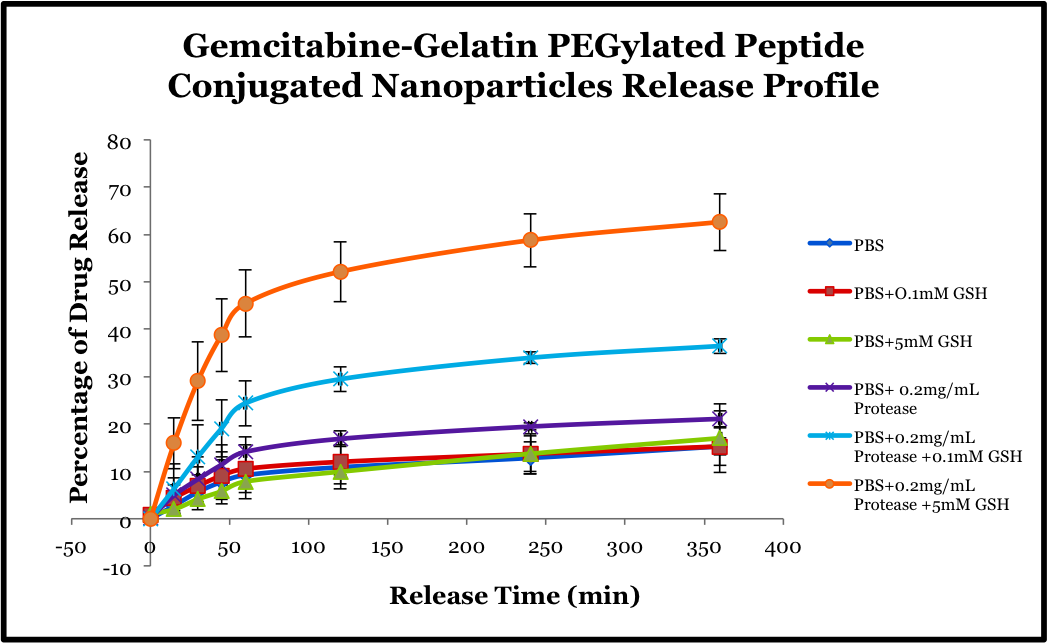

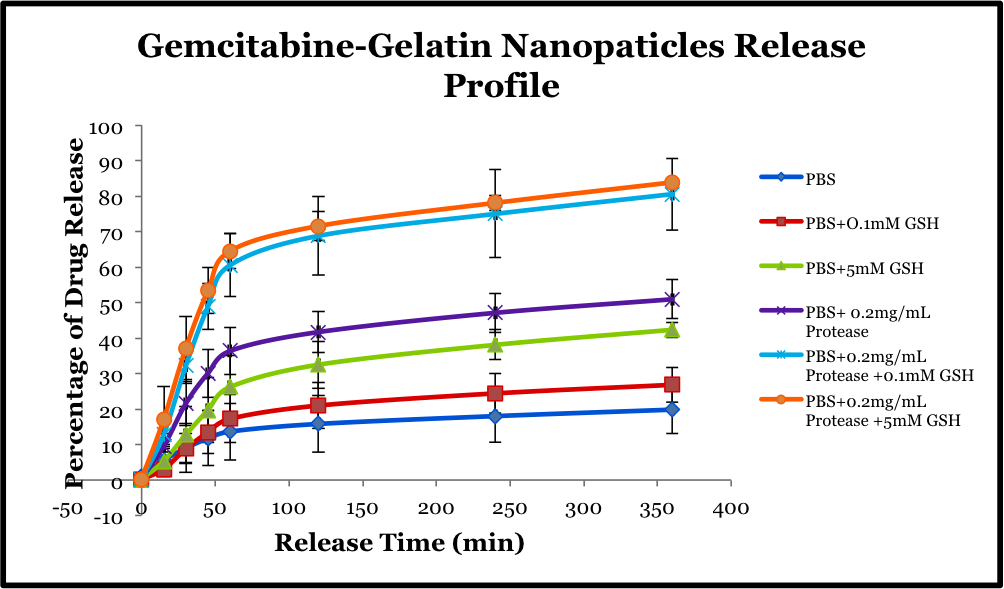

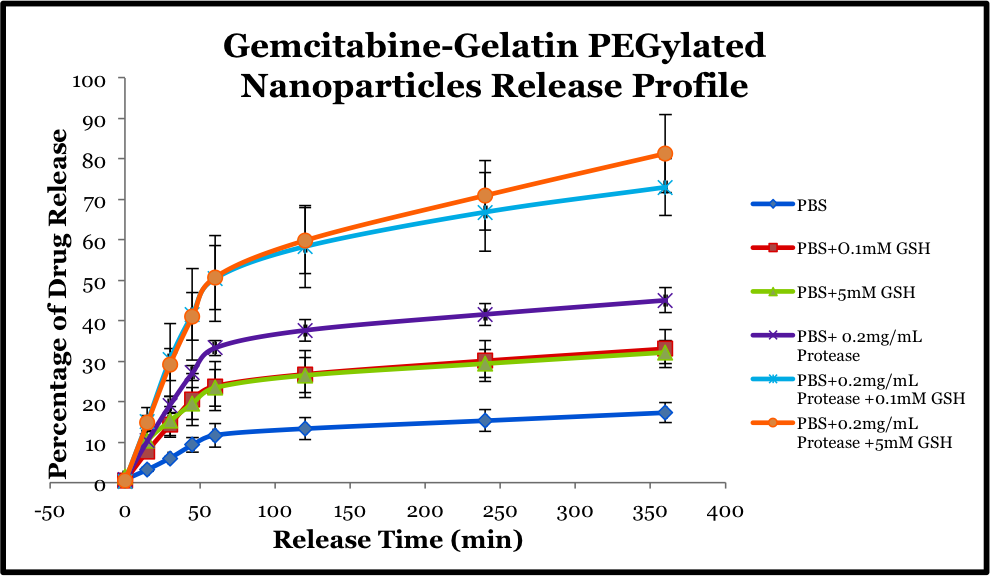


**Figure S1:** *Gemcitabine drug release profile from SH-Gel, SH-Gel-PEG and SH-Gel-PEG-peptide with protease and glutathione in PBS (n=3, Mean ± SD).*

In order to study the release of gemcitabine from gemcitabine-gelatin nanoparticles in biological conditions, protease and glutathione were used to simulate intracellular and extracellular environment. 0.1mM glutathione presented extracellular environment while 5mM glutathione was used to simulate intracellular environment. For unmodified Gemcitabine-Gelatin nanoparticles (SH-Gel), treatments with higher concentration of glutathione could enhance the release of gemcitabine. Protease treatment however presented higher release compared to glutathione treatment alone, which was different form release profile observed from gemcitabine-gelatin polymer. The observation suggests that the structure of nanoparticles hindered the access of glutathione to disulfide bonds that were buried inside nanoparticles. Combined treatment with protease and glutathione could release nearly 90% of the conjugated drug, presenting the best release condition.

Similarly, PEG-modified gemcitabine--gelatin nanoparticles (SH-Gel-PEG), treatment with glutathione enhanced the release of gemcitabine compared to PBS alone. However, higher concentration of glutathione did not change the drug release profile significantly, which might be due to the shielding effect of PEG. Protease treatment showed a better release compared to glutathione treatment while protease-glutathione combination treatment resulted in nearly 80% of the drug release. Interestingly, glutathione treatment did not show any change in release profile compared to PBS in EGFR-targeted nanoparticles (SH-Gel-PEG-peptide), which might be due to the shielding effect of PEG as well as the interaction between glutathione and peptides. Protease treatment presented a slightly better release profile compared to glutathione treatments. Exposure to protease-glutathione combination again resulted in highest drug release from the nanoparticle. The drug release studies confirm that the different gelatin nanoparticle system exhibit a stable morphology in PBS with minimum drug release over time. Most importantly, the intracellular environment mimic resulted in maximum amount of drug release within 6 h, suggesting that these nanoparticles when internalized could successfully release the payload.


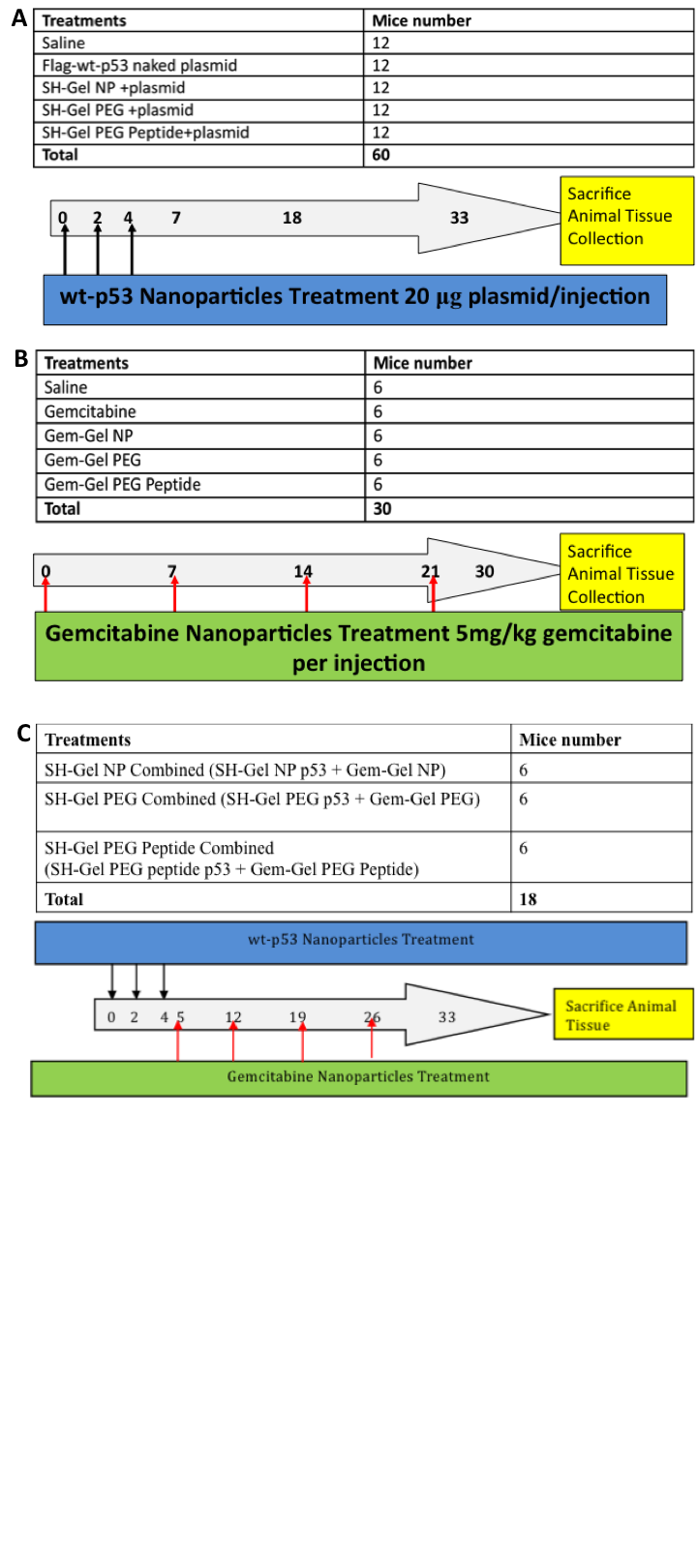


**Figure S2:** *Treatment groups and dose schedule used for (A) p-53 administration, (B) gemcitabine administration and (C) p53-gemcitabine combination adminstration in subcutaneous Panc-1 tumor bearing mice.*


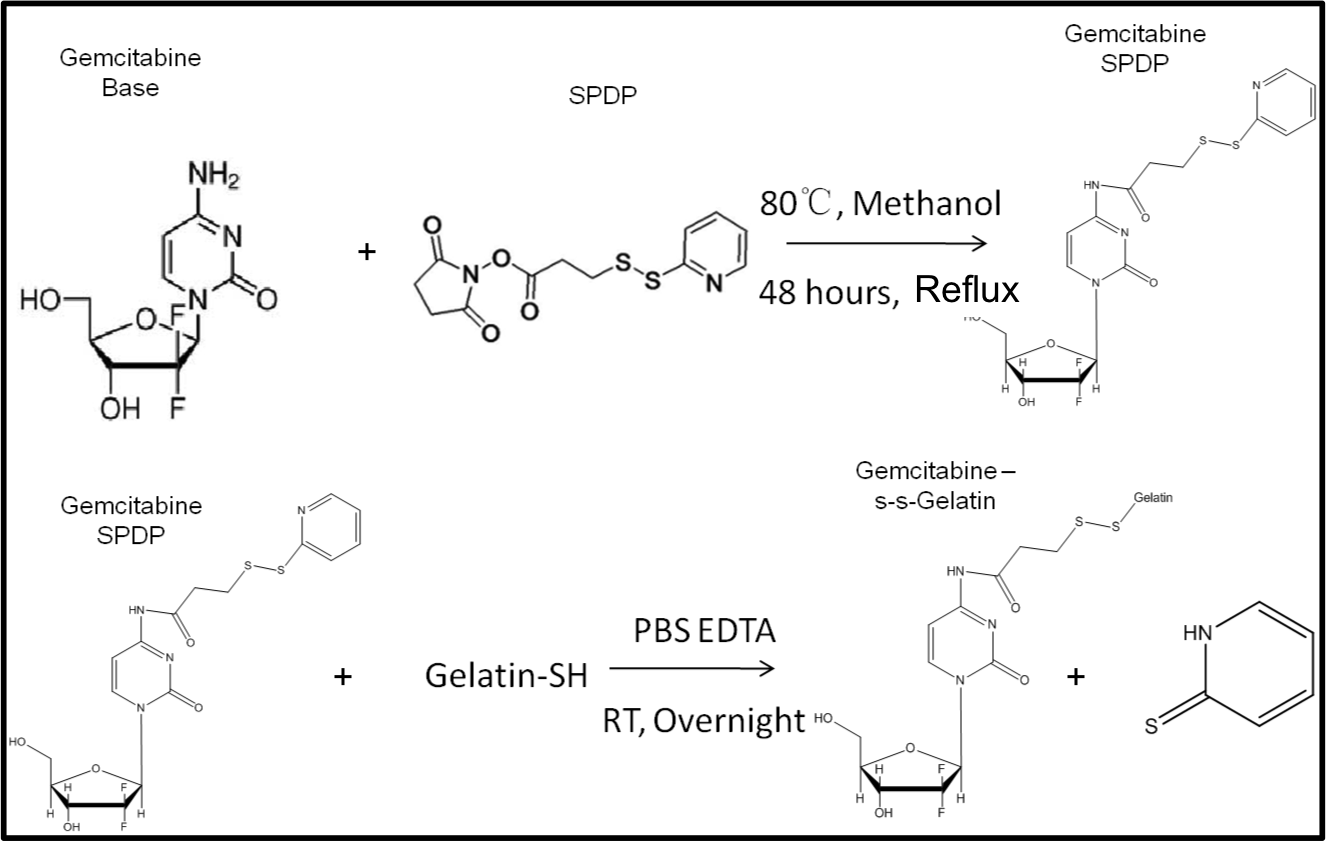


**Figure S3:** *Scheme demonstrating the steps involved in synthesis of gemcitabine conjugated thiolated gelatin.*


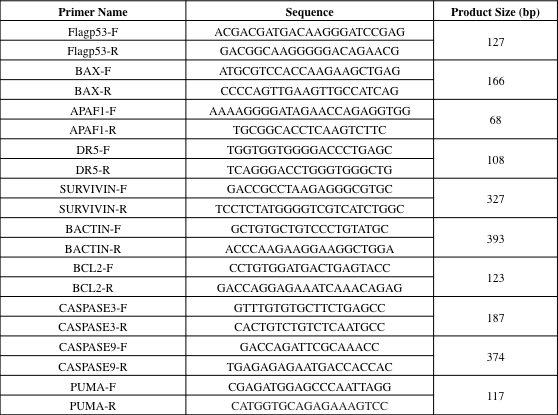


**Table S1:** *Primer sequences used for qPCR analysis of wt-p53, Bax, Apaf-1, DR5, β-actin, Bcl-2, Caspase 3, Caspase 9 and PUMA.*
